# Are physical activity studies in Hispanics meeting reporting guidelines for continuous monitoring technology? A systematic review

**DOI:** 10.1186/s12889-015-2266-4

**Published:** 2015-09-18

**Authors:** Charles S. Layne, Nathan H. Parker, Erica G. Soltero, José Rosales Chavez, Daniel P. O’Connor, Martina R. Gallagher, Rebecca E. Lee

**Affiliations:** Texas Obesity Research Center, Department of Health and Human Performance, University of Houston, Garrison Gymnasium Room 104, 3855 Holman Street, Houston, TX 77204-6015 USA; School of Human Evolution and Social Change, Arizona State University, 900 S. Cady Mall, Tempe, AZ 85287 USA; School of Nursing, University of Texas Health Science Center, 6901 Bertner, Houston, TX 77030 USA; Center for Health Promotion and Disease Prevention, College of Nursing and Health Innovation, Arizona State University, 500 N. 3rd Street, Phoenix, AZ 85004 USA

**Keywords:** Accelerometry, Pedometry, Evidence-based practice, Latinos, Measurement

## Abstract

**Background:**

Continuous monitoring technologies such as accelerometers and pedometers are the gold standard for physical activity (PA) measurement. However, inconsistencies in use, analysis, and reporting limit the understanding of dose–response relationships involving PA and the ability to make comparisons across studies and population subgroups. These issues are particularly detrimental to the study of PA across different ethnicities with different PA habits. This systematic review examined the inclusion of published guidelines involving *data collection*, *processing*, and *reporting* among articles using accelerometers or pedometers in Hispanic or Latino populations.

**Methods:**

English (PubMed; EbscoHost) and Spanish (SCIELO; Biblioteca Virtual en Salud) articles published between 2000 and 2013 using accelerometers or pedometers to measure PA among Hispanics or Latinos were identified through systematic literature searches. Of the 253 abstracts which were initially reviewed, 57 met eligibility criteria (44 accelerometer, 13 pedometer). Articles were coded and reviewed to evaluate compliance with recommended guidelines (*N* = 20), and the percentage of accelerometer and pedometer articles following each guideline were computed and reported.

**Results:**

On average, 57.1 % of accelerometer and 62.2 % of pedometer articles reported each recommended guideline for *data collection*. Device manufacturer and model were reported most frequently, and provision of instructions for device wear in Spanish was reported least frequently. On average, 29.6 % of accelerometer articles reported each guideline for *data processing*. Definitions of an acceptable day for inclusion in analyses were reported most frequently, and definitions of an acceptable hour for inclusion in analyses were reported least frequently. On average, 18.8 % of accelerometer and 85.7 % of pedometer articles included each guideline for *data reporting*. Accelerometer articles most frequently included average number of valid days and least frequently included percentage of wear time.

**Discussion:**

Inclusion of standard collection and reporting procedures in studies using continuous monitoring devices in Hispanic or Latino population is generally low.

**Conclusions:**

Lack of reporting consistency in continuous monitoring studies limits researchers' ability to compare studies or draw meaningful conclusions concerning amounts, quality, and benefits of PA among Hispanic or Latino populations. Reporting data collection, computation, and decision-making standards should be required. Improved interpretability would allow practitioners and researchers to apply scientific findings to promote PA.

## Background

The study of PA has allowed researchers to make significant gains in understanding relationships between PA and related health benefits across a variety of environments and populations [1]. Continuous monitoring technologies such as accelerometers and pedometers have become the gold standard for measurement, and because they are less subjective and uninfluenced by recall, are generally preferred over self-report measures such as questionnaires and surveys [2–7]. Accelerometers measure body movements using piezoelectric sensors and more recently piezoresistive and capacitive technology to monitor acceleration in one to three orthogonal planes [1, 8, 9]. Pedometers are motion sensors worn on the waistband or belt, that use a spring-suspended lever arm that moves concordantly with hip acceleration and deceleration to measure walking distance [10]. Accelerometers are typically preferred over pedometers due to their ability to estimate general PA level and energy expenditure rather than only walking pace and distance [6].

Although continuous monitoring technologies offer more reliable and valid estimates of PA levels, challenges associated with these objective methods such as cost and lack of expertise initially limited their use [6]. Today, technological advances, widespread application, and cost reductions have made accelerometers and pedometers more accessible to researchers interested in assessing PA [6]. As pedometer and accelerometer use has increased, researchers have recognized the need to establish a set of standards and recommendations for procedures involving data collection, processing and reporting of the complex data that are obtained from these continuous monitoring devices. In 2004, the landmark conference ‘Objective Monitoring of PA: Closing the Gaps in the Science of Accelerometry’ addressed inconsistencies in the use, analysis, and interpretation of data obtained from continuous monitoring devices and presented recommendations for achieving standardized use of accelerometers and pedometers [6]. Recommendations included guidelines for the type of device used for specific outcome measures, monitor wear, calibration and sensitivity monitoring, data collection, data filtering and the handling of missing or incomplete data [6]. Many of the concerns expressed by Ward et al. persist today and continue to be debated, indicating that a consensus has not been achieved by PA researchers [11–15]. Lack of consensus and inconsistent reporting limits our ability to make comparisons across studies and understand the dose–response relationship between PA and health outcomes [16]. Non-uniform reporting hampers development of accurate research, policy, and national guidelines such as the recommendations for PA [11, 12].

Although a vexing problem across the realm of PA research, inconsistent standards of data collection, processing and reporting can have a larger impact on the study of PA in specific population subgroups such as Hispanics and Latinos [6]. Significant disparities in PA exist among Hispanics in the U.S. and Latin America. National reports in the US have shown that Hispanics consistently report lower levels (14.4 %) of PA compared to non-Hispanic whites (22.8 %) [17]. International reports have shown that the rate of physical inactivity among Hispanics in Latin America is 43.2 %, reaching as high as 68 % in some countries such as Argentina and Columbia [18]. These low rates of PA have led to the increased prevalence of obesity and development of chronic diseases such as cardiovascular diseases and diabetes in Hispanic populations, especially in low- to middle-income countries in Latin America. The World Health Organization estimates that 80 % of deaths in Latin American countries are attributed to non-communicable diseases [19]. These diseases also carry significant economic consequences, often limiting the social and economic development of the country [20]. Despite these statistics, without standardized practices and reporting guidelines for continuous monitoring devices, researchers are prevented from accurately quantifying PA patterns and related health outcomes in this population [21]. Without standard practices and reporting, researchers are unable to effectively generalize the level of PA and are prevented from making comparisons across studies between Hispanics and other ethnic populations or comparisons within groups of Hispanics [21]. As Hispanics have been identified as a vulnerable population, it is important that standardized practices are implemented and reported for the effective use of continuous monitoring devices in this population.

A systematic review of literature concerning the assessment of PA in Hispanics using continuous monitoring technologies is both timely and warranted. The assessment of PA patterns and their relationships to obesity-related conditions and behaviors in vulnerable populations such as Hispanics has taken on increased importance as preventive models of health care have emerged. This study aimed to compare the reporting of objectively measured PA variables in studies using continuous monitoring devices in Hispanic populations to published, state of the science guidelines about data acquisition, processing, and reporting.

## Methods

### Selection of studies

**Fig. 1:**
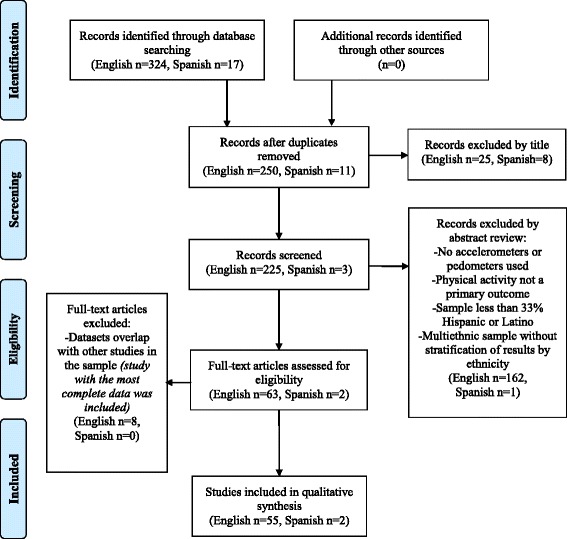
Selection of accelerometer and pedometer studies for systematic review (PRISMA 2009 Flow Diagram)

We systematically identified articles in English or Spanish published between 2000 and 2013 (last search date June 1, 2014) that measured PA among Hispanic or Latino populations of any age living in the United States, Mexico, and South American countries, excluding Brazil as it is economically and culturally different from the other countries that comprise Latin America [21]. We searched PubMed and Ebscohost for English language articles, and we searched SCIELO and Biblioteca Virtual en Salud for Spanish Language articles. Our searches in these databases included the following terms: “Hispanic and accelerometer”, “Mexican and accelerometer”, “Latino and accelerometer”, “Acelometro”, “Hispanic and pedometer”, “Mexican and pedometer”, “Latino and pedometer” and “pedometro” [22]. Search terms identified studies including Mexican-Americans, as this is the largest and most prevalent group of Hispanics in the U.S. [23]. This search and an examination of titles produced a total of 228 articles (225 in English, 3 in Spanish), which underwent initial abstract review for use of continuous monitoring technologies (accelerometers and/or pedometers). Articles were further screened for the following criteria: 1) PA as a primary outcome, 2) sample included at least 33 % Hispanic or Latino, and, for articles including multiethnic samples, 3) stratification of continuous monitoring results for ethnicity. For articles on which two reviewers disagreed regarding inclusion, the reviewers discussed rationale for inclusion or exclusion and referred one another to evidence in the text until consensus was reached. Sixty-five articles (63 in English, 2 in Spanish) met all inclusion criteria and underwent final review. If multiple articles reported results from the same study or dataset, the article including the most complete accelerometry data was included in analyses. Eight articles were eliminated accordingly, leaving 57 articles (55 in English, 2 in Spanish) for analyses. Figure 1 presents the number of articles identified, the number of articles included and excluded, and the rationale for inclusion or exclusion for each step of the systematic search.

### Development and application of reporting guidelines

**Fig. 2:**
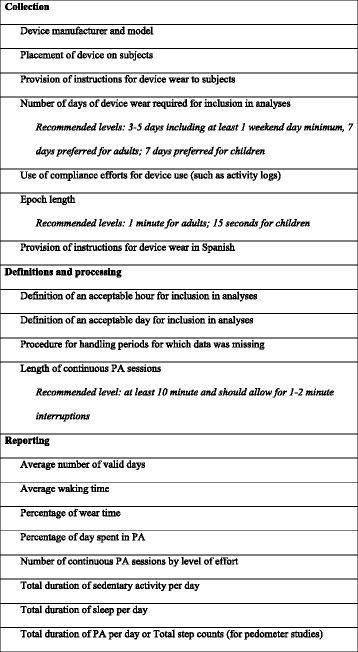
Gold standards in accelerometry and pedometry data reporting

The purpose of this systematic review was to compare the reporting of continuous monitoring variables in the PA literature involving Hispanic samples to the published guidelines regarding data collection, processing and reporting of continuous PA monitoring. Eighteen reporting guidelines were extracted from the 2005 article by Ward et al. outlining areas for researchers to improve the validity of data collected using accelerometers in studies of PA [6]. Each of the guidelines included in this review was identified by Ward et al. as a “best practice” in continuous monitoring data collection that, with standardized reporting, facilitates data analysis and cross-study comparisons. Two reporting guidelines were added for applicability to studies involving Hispanic or Latino populations (“Provision of instructions for device wear in Spanish”) and studies using pedometers (“Total step counts”). All reporting guidelines are presented in Fig. 2.

Among the 20 reporting guidelines applied to the articles reviewed, 7 were parameters governing data collection, 4 were parameters governing data definitions and processing, and 9 were reporting standards for summary variables (Fig. 2). All articles were coded with “yes” or “no” depending on whether they reported each guideline. The Ward, et al. article establishes recommended levels at which three of the reporting guidelines should be set in studies involving continuous PA monitoring: number of days of device wear required for inclusion in analyses, epoch length, and length of continuous PA sessions [6]. For these guidelines, articles were coded for both inclusion (yes/no) and the actual level applied to data collection or data inclusion (e.g. collecting accelerometry data in epoch lengths of 1 min and processing data into continuous PA sessions of 10 min or longer) (Fig. 2).

### Collection of descriptive and contextual information

In addition to the 20 reporting guidelines for continuous monitoring technologies, coders recorded identifying, descriptive, and contextual information from each article. The six identifying characteristics collected included article authors, article title, journal, year, volume/issue, and page numbers. The 10 general descriptive and contextual characteristics collected included, when applicable and described in articles, the name of trial or study, type of study, a description of the study’s intervention, groups by which data were reported, subject ethnicities, inclusion criteria, study location, study environment (urban/suburban by default, rural when stated explicitly by authors), intervention setting, and assessment setting. The final five descriptive and contextual characteristics applied only to the Hispanic or Latino subjects for whom continuous monitoring variables were reported: number of participants, gender, number of females, number of overweight or obese, and age.

### Coding protocol

Ten trained coders participated in article coding. Training was completed in two sessions. In the first training session, coders were given a codebook that listed all reporting guidelines and contextual variables. The codebook provided operational definitions for each code as well as examples of plausible answers that were likely to be reported in the literature. Coders were trained on how to complete each coding category on the coding sheet using definitions from the codebook. Coders were instructed to complete each category with “yes” to indicate the inclusion of a reporting guideline or “no” to indicate the absence of a reporting guideline. For the three guidelines involving set recommended levels (Fig. 2), coders were also instructed to find and record applicable data for these categories. For contextual variables, coders were only instructed to find and record applicable data. Following the first training, coders were divided into pairs and asked to independently code three randomly selected articles for the inclusion of reporting guidelines. In the second training session, coding pairs met to review and compare their coding sheets for the initial three articles. Discrepancies were resolved by referring to the codebook and were discussed as a group to ensure understanding among all coding pairs. Following this training, each member of coding pairs independently coded a list of randomly assigned articles for the presence or absence of the reporting guidelines listed in Fig. 2 and for applicable data regarding the three reporting guidelines with recommended levels. Spanish language articles were assigned to pairs in which both coders could fluently speak and read Spanish. After coding was completed, coding pairs met to review and compare coding sheets for all coded articles. Each pair recorded the number of disagreements and agreements for each coding sheet. These values were used to analyze inter-rater reliability (*n* = 28). Inter-rater reliability was high (Kappa = 0.804). Coders then discussed disagreements and resolved them by consensus, referring one another to data in research articles for resolution. Once a consensus was reached, one master coding sheet for each article was submitted for analysis.

## Results

### Characteristics of studies

Contextual information for each of the articles reviewed is listed in Table 1. Of the 57 articles included and coded, 44 utilized accelerometers and 13 utilized pedometers. Approximately 75 % of studies were cross-sectional, and among the 14 longitudinal studies, 13 (93 %) involved interventions. Sixty-three percent of studies included solely Hispanic or Latino participants, and 37 % of studies involved multiethnic samples. Eighty-nine percent of studies were conducted within the United States, and 9 % were conducted in Mexico. Eighty-two percent of studies occurred in urban settings, 11 % involved participants in both urban and rural settings, and 7 % occurred in rural settings. On average, study samples included 203 Hispanic or Latino participants (range 8–1248) who were 73 % female (range 13.8–100 %), and 29 years old (range 3.7–73.7).

**Table 1:** Article characteristics

Author and year	Study design	Sample ethnicity	Country	Setting	Hispanic or Latino participants (n)	Female (%)^a^	Mean age (years)^a^
*Accelerometer articles*							
Ainsworth et al., 2013 [24]	Cross-sectional	Hispanic or Latina	USA	Urban	139	100.0	28.3
Alhassan et al., 2007 [25]	Cross-sectional	Latino	USA	Urban	32	37.5	3.7
Boudreau et al., 2013 [26]	Intervention	Latino	USA	Urban	41	61.5	-
Bacardí-Gascón et al.,2004 [27]	Cross-sectional	Mexican	Mexico	Rural	100	100.0	53.0
Bacardí-Gascón et al., 2011 [28]	Cross-sectional	Mexican	Mexico	Urban	35	51.0	4.4
Bennett et al., 2006 [29]	Cross-sectional	Multiethnic	USA	Urban	182	64.0	-
Butte et al., 2007 [30]	Cross-sectional	Hispanic	USA	Urban	897	51.0	10.8
Byrd-Williams et al., 2007 [31]	Cross-sectional	Multiethnic	USA	Urban	106	50.0	9.4
Byrd-Williams et al., 2010 [32]	Intervention	Hispanic	USA	Urban	38	50.0	-
Casazza et al., 2009 [33]	Cross-sectional	Hispanic-American	USA	Urban	55	-	-
Christensen et al., 2012 [34]	Cross-sectional	Mexican	Mexico	Rural	64	63.0	40.7
Clarke et al., 2007 [35]	Intervention	Multiethnic	USA	Urban	69	100.0	27.0
Davis et al., 2011 [36]	Intervention	Hispanic or Latina	USA	Urban	38	100.0	15.8
Evenson et al., 2012 [37]	Cross-sectional	Multiethnic	USA	Urban and rural	530	50.0	70.0
Gay et al., 2013 [38]	Cross-sectional	Multiethnic	USA	Urban	118	68.6	48.0
Godard et al., 2012 [39]	Cross-sectional	Hispanic or Latino	Chile	Urban	109	38.5	-
Gortmaker et al., 2012 [40]	Cross-sectional	Multiethnic	USA	Urban and rural	1248	-	-
Ham et al., 2010 [41]	Cross-sectional	Multiethnic	USA	Urban and rural	658	51.5	-
Hennessy et al., 2010 [42]	Cross-sectional	Multiethnic	USA	Rural	19	65.8	9.1
Holman et al., 2011 [43]	Cross-sectional	Multiethnic	USA	Urban and rural	1038	49.2	13.0
Hoos et al., 2012 [44]	Cross-sectional	Hispanic or Latina	USA	Urban	71	100.0	43.0
Kligerman et al., 2006 [45]	Cross-sectional	Mexican-American	USA	Urban	60	27.0	16.2
Koniak-Griffin et al., 2013 [46]	Cross-sectional	Mexican-American	USA	Urban	223	100.0	44.6
Layne et al., 2011 [47]	Cross-sectional	Multiethnic	USA	Urban	71	100.0	46.6
Lee et al., 2012 [48]	Cross-sectional	Multiethnic	USA	Urban	148	100.0	41.4
Lohman et al., 2006 [49]	Cross-sectional	Multiethnic	USA	Urban	337	100.0	12.0
Lovasi et al., 2011 [50]	Cross-sectional	Multiethnic	USA	Urban	355	-	-
Marquez et al., 2008 [51]	Cross-sectional	Hispanic or Latino	USA	Urban	148	83.0	29.4
Marquez et al., 2011 [52]	Cross-sectional	Hispanic or Latino	USA	Urban	174	73.6	27.4
Marshall et al., 2013 [53]	Intervention	Hispanic or Latina	USA	Urban	180	100.0	36.9
McClain et al., 2011 [54]	Cross-sectional	Multiethnic	USA	Urban	40	100.0	9.4
Medina et al., 2013 [55]	Cross-sectional	Mexican	Mexico	Urban	262	48.3	37.2
Mendoza et al., 2013 [56]	Cross-sectional	Hispanic or Latino	USA	Urban	96	40.0	4.7
Hernandez et al., 2013 [57]	Cross-sectional	Mexican	Mexico	Urban	71	-	-
Nicaise et al., 2011 [58]	Cross-sectional	Hispanic or Latina	USA	Urban	105	100.0	35.9
Olvera et al., 2011 [59]	Longitudinal	Hispanic or Latina	USA	Urban	102	100.0	36.0
Ramirez-Marrero et al., 2008 [60]	Cross-sectional	Hispanic or Latino	USA (Puerto Rico)	Urban	58	39.6	46.5
Ruiz et al., 2011 [61]	Intervention	Hispanic or Latino	USA	Urban	212	-	-
Sanchez et al., 2007 [62]	Cross-sectional	Multiethnic	USA	Urban	115	13.8	-
Schaefer et al., 2013 [63]	Intervention	Hispanic or Latino	USA	Rural	170	-	-
Spruijt-Metz et al., 2009 [64]	Cross-sectional	Hispanic or Latina	USA	Urban	10	100.0	12.1
Trost et al., 2012 [65]	Cross-sectional	Multiethnic	USA	Urban	251	-	-
Vella et al., 2011 [66]	Cross-sectional	Hispanic or Latina	USA	Urban	60	100.0	25.2
Wilbur et al., 2012 [67]	Cross-sectional	Hispanic or Latino	USA	Urban	174	73.6	66.0
*Pedometer articles*							
Bender et al., 2013 [68]	Intervention	Hispanic	USA	Urban	33	76.0	15.3
Coffman et al., 2013 [69]	Intervention	Latina	USA	Urban	27	100.0	47.0
D’Alonzo et al., 2004 [70]	Intervention	Multiethnic	USA	Urban	8	100.0	26.5
D’Alonzo et al., 2007 [71]	Cross-sectional	Hispanic or Latina	USA and Costa Rica	Urban	17	100.0	14.7
Dauenhauer et al., 2011 [72]	Cross-sectional	Multiethnic	USA	Urban	54	53.5	9.8
Drieling et al., 2013 [73]	Cross-sectional	Hispanic or Latino	USA	Urban	207	76.8	31.1
Hernandez et al., 2013 [74]	Intervention	Hispanic or Latino	USA	Urban	572	77.1	73.7
Johnson et al., 2010 [75]	Cross-sectional	Multiethnic	USA	Urban and rural	308	53.1	10.4
Keller et al., 2011 [76]	Cross-sectional	Hispanic or Latina	USA	Urban	271	100.0	55.5
Kulinna et al., 2012 [77]	Intervention	Multiethnic	USA	Urban and rural	271	48.1	9.7
Oh et al., 2012 [78]	Cross-sectional	Hispanic or Latino	USA	Urban	101	58.0	14.8
Pekmezi et al., 2013 [79]	Intervention	Hispanic or Latina	USA	Urban	43	100.0	41.6
Shelton et al., 2009 [80]	Cross-sectional	Multiethnic	USA	Urban	680	-	-

### Guidelines for data collection

The initial set of guidelines reviewed were standards pertaining to data collection. Percentages of articles reporting guidelines for data collection are listed in Table 2. A majority of articles (89 % of accelerometry studies and 93 % of pedometry studies) reported the device manufacturer and model. Our review found that 73 % of accelerometry articles and 40 % of pedometry articles reported device placement (e.g. on the left hip). Slightly more than half (59 %) of the accelerometry articles and 80 % of pedometry articles reported providing the participants with specific instructions concerning wearing the device (e.g. remove during bathing). Sixty-six percent of accelerometry articles and 73 % of pedometry articles reported the number of days of device wear that were required to include participants in analyses. Our review found that only 21 % of accelerometry articles and 47 % of pedometry articles reported measures or indicators of compliance with device use, such as the use of PA logs or reminders via email or phone. Very few (11 % of accelerometry articles and 40 % of pedometry articles) articles reported providing instructions for device wear in Spanish. Reporting on epoch length was fairly high, with 82 % of accelerometry articles reporting the preset epochs at which PA data were sampled.

**Table 2:** Percentages of articles reporting guidelines for continuous monitoring data

Guideline	Accelerometer articles (*n* = 44) (%)	Pedometer articles (*n* = 13) (%)
*Data collection standards*		
Device manufacturer and model	88.6	92.9
Placement of device on subjects	72.7	40.0
Provision of instructions for device wear to subjects	59.1	80.0
Number of days required for inclusion in analyses	65.9	73.3
Use of compliance efforts for device use	20.5	46.7
Provision of instructions for device wear in Spanish	11.4	40.0
Epoch length	81.8	-
*Data inclusion standards*		
Definition of an acceptable hour for inclusion in analyses	6.8	-
Definition of an acceptable day for inclusion in analyses	75.0	-
Procedure for handling periods for which data was missing	11.4	-
Length of continuous PA sessions	25.0	-
*Data reporting standards*		
Average number of valid days	56.8	-
Average waking time	9.1	-
Percentage of wear time	2.3	-
Percentage of day spent in PA	22.7	-
Number of continuous PA sessions by level of effort	13.6	-
Total duration of sedentary activities per day	25.0	-
Total duration of sleep per day	4.6	-
Total duration of PA per day	15.9	-
Total step count	-	85.7

### Guidelines for data definitions and processing

Additional guidelines published by Ward et al. [6] recommended standardized data definitions and processing procedure. Table 2 lists percentages of articles that reported following these guidelines. Only 7 % of accelerometry articles reported their definition of an acceptable hour (i.e. the number of minutes in which a predetermined level of ‘counts’ were registered by the accelerometer for that hour to be included in the data analyses). The investigator’s definition of an acceptable day (i.e. the amount of absolute time or a percentage of a measurement period that a device was worn for the data from that day to be included in the data analyses), was reported more frequently, with 75 % of articles reporting this aspect. As there are often compliance issues with participants wearing continuous monitoring devices throughout the sample period, it is important to report the procedure for handling missing data. In this study, only 11 % of articles reported the procedure used for handling periods of missing data. Ward, et al. [6] recommend that researchers report the number of wear interruptions (lengths of time during which no activity was recorded) and utilize imputation to replace missing data. However, this information was reported in only 25 % of accelerometer articles.

### Guidelines for data reporting

The remaining nine guidelines that were examined were standards for data reporting. Data for these standards can be calculated and obtained by continuous monitoring devices and their associated software providing summaries of many variable that are useful for measuring and monitoring PA outcomes [6]. Percentages of articles reporting these standards are listed in Table 2. Only 57 % of accelerometry articles reported the average number of valid days of wear. Only 9 % of accelerometry articles reported the average waking time of participants. The percentage of wear time was reported in only 2 % of accelerometry articles. The percentage of the day spent in PA was reported in 23 % of accelerometry articles while the number of continuous PA sessions by level of effort was reported in 14 % of accelerometry articles. The total duration of sedentary activities per day was reported in 25 % of articles. Finally, the total duration of sleep per day was reported in only one accelerometry article (5 %). The total duration of PA per day was reported in just 16 % of accelerometry articles, but total pedometer step counts were reported in 86 % of pedometry articles.

## Discussion

This purpose of this systematic review was to compare the reporting of continuous monitoring variables in studies that included Hispanic populations to published guidelines on data acquisition, processing and reporting. These guidelines were published in an effort to promote the standardization of future data acquisition, processing, and data reporting across studies using continuous monitoring devices. Findings from this review confirm that many of the issues identified by Ward et al. [6] in data collection, processing, and reporting still persist today, and are particularly prevalent in studies using continuous monitoring devices in Hispanic populations. Slightly over half of the articles reviewed met standards for data collection, with only a third of articles meeting standards for data processing. The majority of pedometer articles met standards for data reporting; however, less than 20 % of accelerometer articles met these same standards [11–15].

The first set of guidelines reviewed were standards pertaining to data collection. The majority of studies reported the make and model of the device used. Reporting the make and model of the device is important for considerations of practicality, cost, monitor compatibility, and the reliability and validity of reported data [6]. Reporting on device placement was also high among accelerometer and pedometer articles. Since the location for device placement on subjects may vary by subject demographics (e.g. age) and affects data outputs, this information is important for helping readers interpret the published data [6]. The majority of articles reported providing instructions on device wear to participants; however, very few accelerometer articles and less than half of pedometer articles reported providing these instructions in Spanish. Efficient and reliable data collection requires that investigators provide and participants comply with clear instructions for device wear, and failure to do so can result in misleading and inaccurate data [6]. Failure to provide instructions in the participant’s preferred language may lead to miscommunication that could negatively impact data collection processes.

The majority of articles in this review reported on device wear, indicating the number of days of wear required for participants to be included in analyses so that PA patterns could be more accurately estimated. The number of days of device wear is also an important component of monitoring protocols and may vary based on study setting, subject demographics, research questions, and study resources. According to Ward et al., 7 day monitoring protocols with at least 3–5 days of monitoring is needed in order to estimate habitual PA among adults and children [6]. Few accelerometer articles and half of pedometer articles reported the use of compliance efforts such as activity logs and phone call reminders to participants. Use of these compliance efforts for device use can also help researchers obtain accurate estimates of habitual PA, adding to the interpretability of PA findings by providing specific information on activities and location of activity [6]. For preset sampling periods, or epochs, Ward et al. recommend 1-min sampling epochs for adults and 15-s sampling epochs for children. Almost all accelerometer articles met the guideline for reporting preset epochs. This guideline is important for accurate measurement of PA since different epoch lengths can result in different estimates of moderate and vigorous PA.

The second set of guidelines published by Ward et al. [6] outlined data definitions and processing standards. The first standard addressed criteria for the constitution of time periods for device wear. Defining the amount of device wear that constitutes an acceptable hour of wear for inclusion in analyses is important, as short durations of device wear may provide less accurate estimates of habitual PA. For accurate interpretation of data, the definition of an acceptable hour should be standardized and applied uniformly to all study participants; however, only 7 % of accelerometer articles reported the definition of an acceptable hour used by the investigators. The handling of missing data is another important guideline for data processing. Only 11 % of articles reported a procedure for handling missing data. Missing data for sampling periods can significantly skew the data, causing PA measurements to be severely over- or underestimated [6]. Reporting on the number and length of continuous sessions in which PA occurs was also poor. The number and length of continuous sessions lends to the interpretation of data and definition PA patterns and can also have important health implications for the study population [6].

The remaining nine guidelines examined were standards for data reporting. Effective comparisons between studies require the standardized reporting of PA variables. However, few of these standards were reported in the articles included in this review. Only 10 % of articles reported information on participant use such as average waking time and percentage of wear time. Other PA variables such as duration of PA per day, percentage of the day spent in PA, the number of continuous PA sessions by level of effort, the total duration of sedentary activities and duration of sleep time per day were also underreported. The only standard consistently reported in the majority of pedometer articles was the total pedometer step counts.

Findings from this study have important implications for research and practice. Based on the results of our analyses, few investigators report the information necessary to be compliant with all of the recommended guidelines for data collection, processing and reporting of PA obtained from continuous monitoring technologies. Researchers should include instructions in the native or preferred language of the population they are targeting. Non-English speaking and/or immigrant minorities may be apprehensive of continuous monitoring devices. Providing instructions in their native language will serve to build trust between participants and the research team, promotes compliance, and ensures that devices are worn appropriately and that their use and placement is understood by participants. Furthermore, standards on data definitions and processing were poorly reported. Many investigators rely on previously conducted studies in order to compile evidence and make comparisons of outcomes across studies and populations. However, this review has revealed that it is very often unclear how previous researchers collected and processed their data, potentially resulting in misinterpretations and inaccurate use of PA. This can further perpetuate beliefs about PA patterns that are unfounded and can misinform policy recommendations. Likewise, researchers must increase their reporting of the data standards they employed in their investigations. This information is also critical in allowing researchers to compare PA outcomes across studies in order to increase our understanding of PA behaviors in specific populations. Researchers who use continuous monitoring devices must make a stronger effort to follow and report the guidelines provided by Ward et al. [6] and outlined in this study.

Journals and the scientific community would benefit greatly from requiring authors of papers involving continuously monitored PA to submit standardized tables or appendices listing (1) all data collection strategies they utilized, (2) all standards by which decisions to include or exclude data from analyses were made, and (3) standard variables that can be computed from their datasets. We recommend that these standards be followed and reported to make the data and use of these devices meaningful and generalizable. Improving the reporting of standards can provide important information that can help fight ethnic disparities in PA among Hispanic populations.

## Conclusion

Lack of consensus and inconsistent reporting limits our ability to make comparisons across studies and limits our understanding of the dose–response relationship between PA and health outcomes, particularly in special populations such as Hispanics [16]. Failure to achieve uniformity in reporting can leave future research, policy, and national guidelines, such as recommendations for PA and strategies to improve compliance in PA interventions, misinformed [1, 12]. Findings from this study showed that, in studies using continuous monitoring devices in Hispanic populations, reporting on data collection methods is inconsistent and reporting on methods and definitions for data processing is poor. Failure to follow standard guidelines for data collection, processing, and reporting has a number of consequences. First, these failures prohibit effective generalization of the level of physical activity for Hispanics from a particular sample and study to the population. Second, these failures may produce inaccuracies in monitoring and tracking of PA patterns and related health outcomes, which may prevent us from effectively intervening in this population. Finally, these omissions limit our ability to make comparisons across studies between Hispanics and other ethnic populations or comparisons within varying groups of Hispanics, which can provide additional insights on PA trends and disparities [81].

## Abbreviation

PA: Physical activity
